# Supplementary material for: Label-free chemical imaging of cytochrome P450 activity by Raman microscopy
Source: Commun Biol. 2022 Aug 22;5:778. doi: 10.1038/s42003-022-03713-1 (PMC9395422; doi:10.1038/s42003-022-03713-1)
Supplement: Supplementary file 2 — Supplementary Information [file 42003_2022_3713_MOESM2_ESM.pdf]

Supplementary Information

Label-free chemical imaging of cytochrome P450 activity by Raman Microscopy

Menglu Li[1,2†], Yasunori Nawa[1,2†], Seiichi Ishida[1,3,4], Yasunari Kanda[1,4], Satoshi Fujita[1,2*], Katsumasa Fujita[1,2,5*]

AFFILIATIONs:

[1] AIST-Osaka University Advanced Photonics and Biosensing Open Innovation Laboratory, National Institute of Advanced Industrial Science and Technology (AIST), 2-1 Yamadaoka, Suita, Osaka 565-0871, Japan.

[2] Department of Applied Physics, Osaka University, 2-1 Yamadaoka, Suita, Osaka 565-0871, Japan.

[3] Division of Applied Life Science, Graduate School of Engineering, Sojo University, 4-22-1, Ikeda, Nishi-ku, Kumamoto 860-0082, Japan.

[4] Division of Pharmacology, National Institute of Health Sciences, Kawasaki, Kanagawa 210-9501, Japan.

[5] Institute for Open and Transdisciplinary Research Initiatives, Osaka University, 2-1 Yamadaoka, Suita, Osaka 565-0871, Japan.

* Correspondence to: Satoshi Fujita, Katsumasa Fujita

† Equal contributions

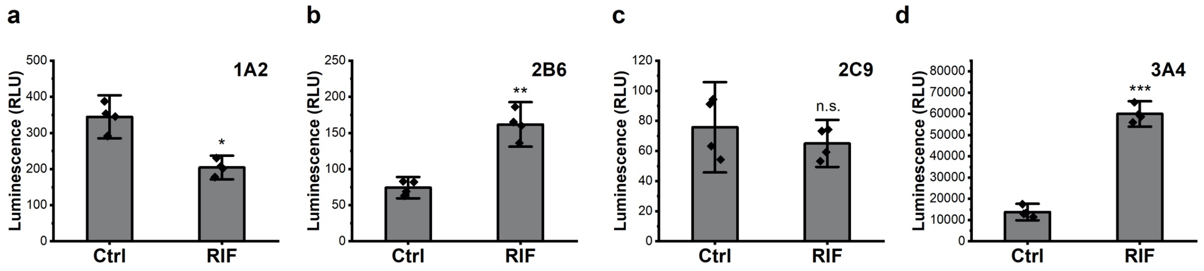

Supplementary Fig. 1 Changes in CYP activity after treatment with RIF. a 1A2 activity. **b** 2B6 activity. **c** 2C9 activity. **d** 3A4 activity.

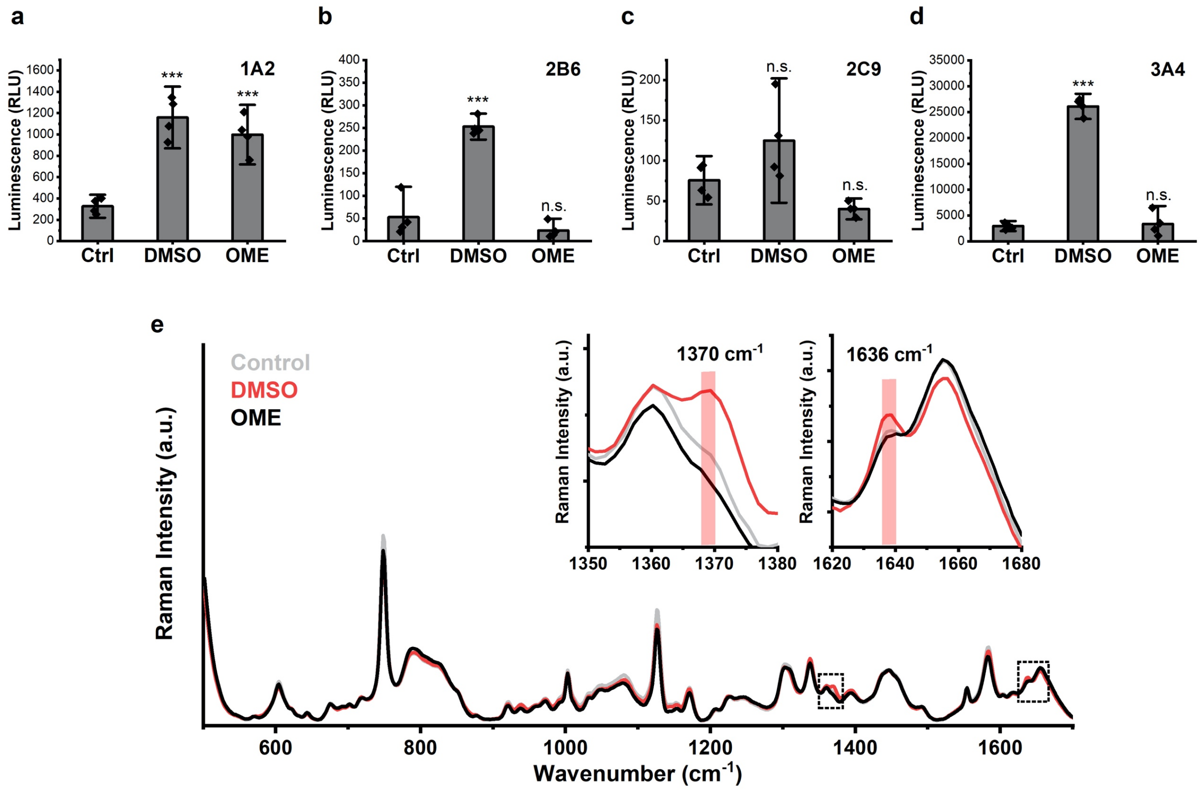

Supplementary Fig. 2 Raman measurement of CYP activity induced by DMSO and omeprazole (OME). a 1A2 activity. **b** 2B6 activity. **c** 2C9 activity. **d** 3A4 activity. e Raman spectra of control, DMSO, and OME treated HepaRG cells. Raman peaks at 1370 cm^{-1} and 1636 cm^{-1} were enlarged and shown as inserts. A significant increase was detected at 1370 cm^{-1} and 1636 cm^{-1} in DMSO-treated HepaRG cells but not in OME-treated cells.

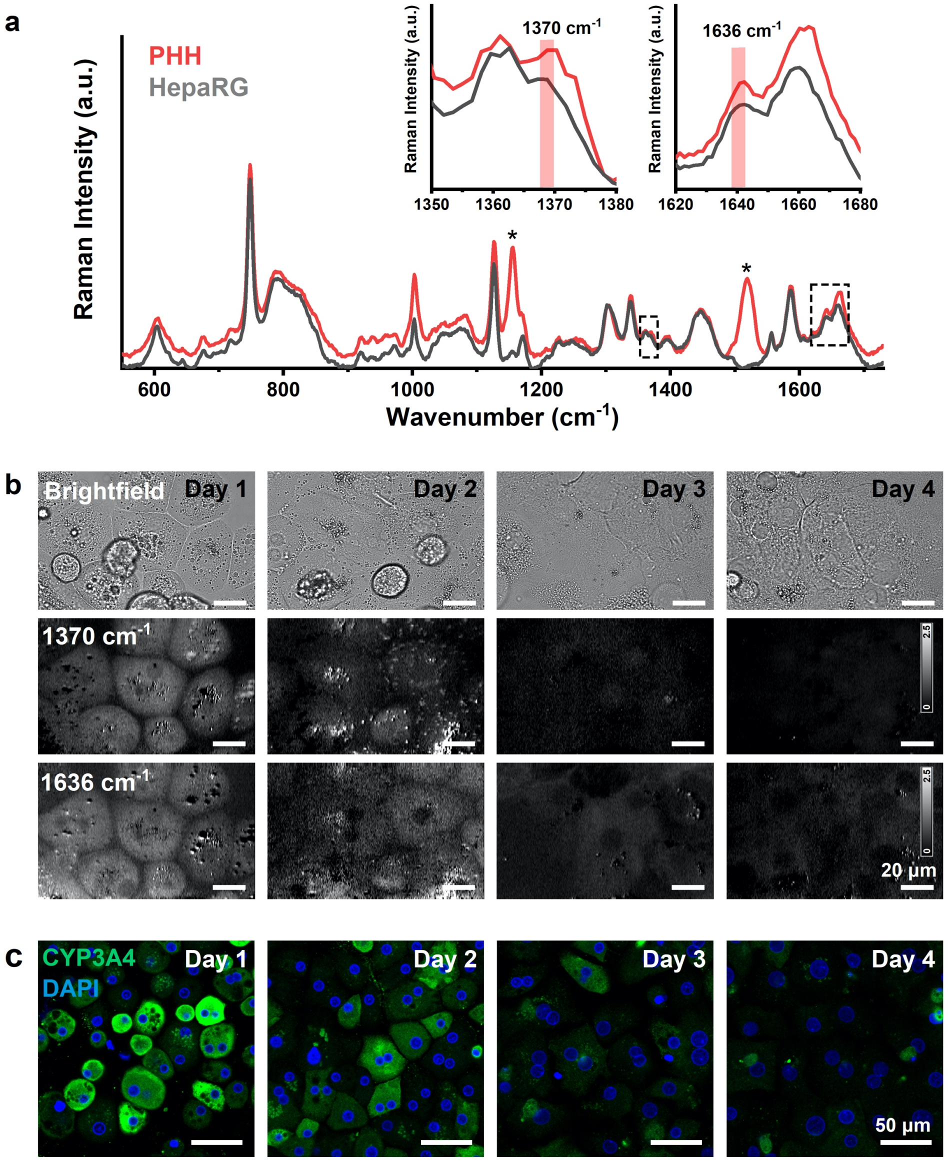

Supplementary Fig. 3 Raman measurement of primary human hepatocytes (PHHs). a Raman spectra of PHH cells (red) and HepaRG cells (gray) were taken after the cells were fully attached to the culture surface. The Raman peaks at 1370 $cm^{-1}$and 1636 cm^{-1} were enlarged and shown in the inserts. Asterisks indicate Raman peaks (1157 cm^{-1} and 1512 cm^{-1}) detected in PHH but not in HepaRG cells, which can be assigned to carotenoids stored in the liver. **b** The reconstructed Raman images at 1370 cm^{-1} and 1636 cm^{-1}, which can be assigned to oxidized and low-spin heme, respectively. A time-dependent decrease in Raman signal was observed. Scale bars, 20 μm. **c** Immunofluorescence staining of CYP3A4 and nuclear counterstain by DAPI. Scale bars, 50 μm.

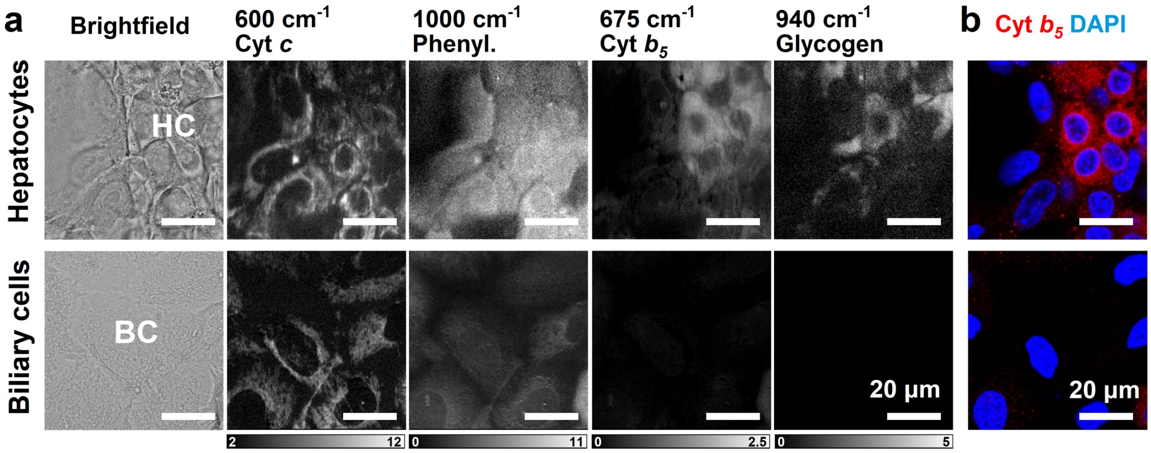

Supplementary Fig. 4 Reconstructed Raman images of Hepatocyte and biliary cells and in situ immunofluorescence staining of cyt *b*$_5$. a Raman images at 600 cm^{-1}, 1000 cm^{-1}, 675 cm^{-1}, and 940 cm^{-1}, which can be assigned to cyt *c*, phenylalanine, cyt *b*$_5$, and glycogen, respectively. Scale bars, 20 µm. **b** Immunofluorescence staining of cyt *b*$_5$ with nuclear counterstain by DAPI. Scale bars, 20 µm. The stained images show that more cyt *b*$_5$ is expressed in hepatocytes than in biliary cells.

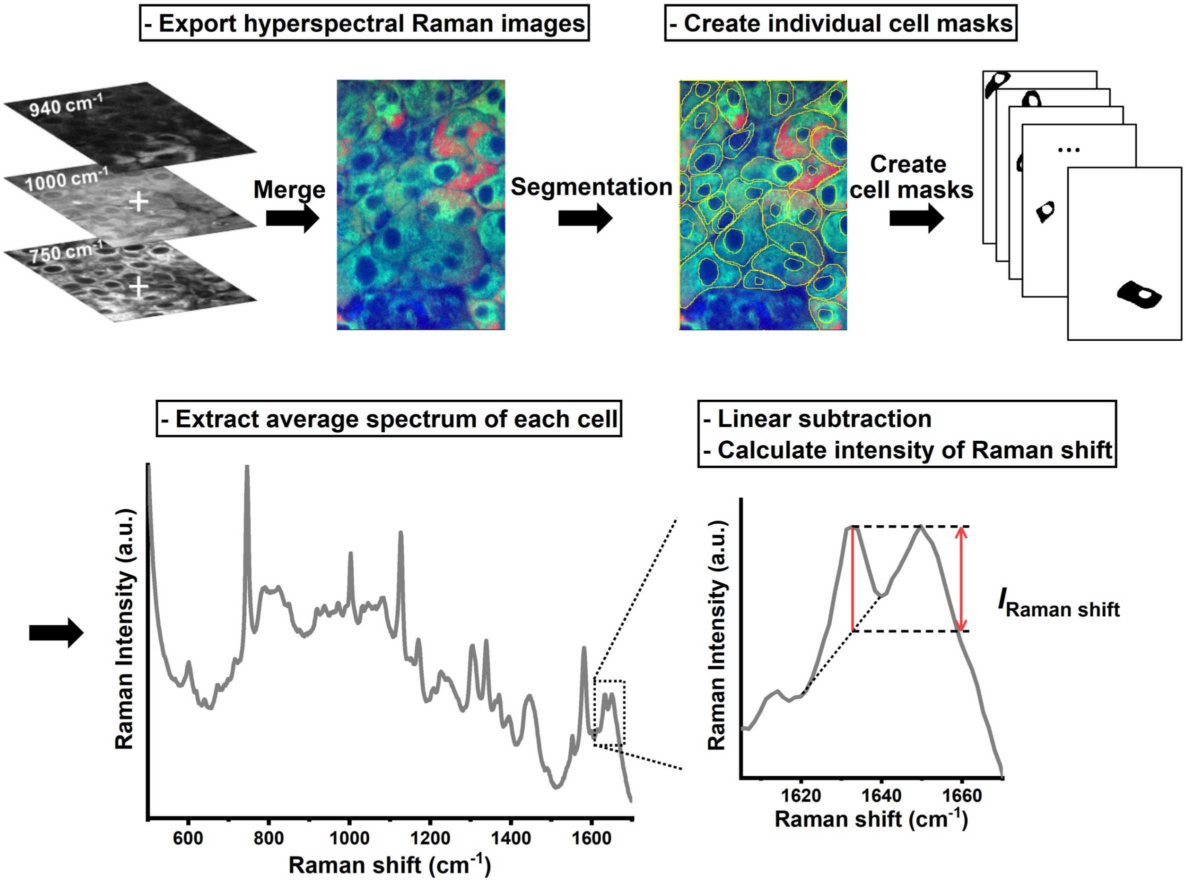

Supplementary Fig. 5 Single cell-based quantification of Raman intensity. Hyperspectral Raman images were constructed, and images at 750 cm^{-1}, 940 cm^{-1}, and 1000 cm^{-1} were chosen to present cell boundaries and nuclear shapes. The segmentation of the cytoplasm was performed manually by subtracting the nucleus from the cell body. The cell masks were then applied to the raw data after cosmic-ray removal (without SVD to ensure the quantitative feature of Raman spectroscopy) for calculating the average spectrum of each cell. The Raman intensity of the target shifts was calculated after baseline correction to reduce interference from adjacent Raman shifts.

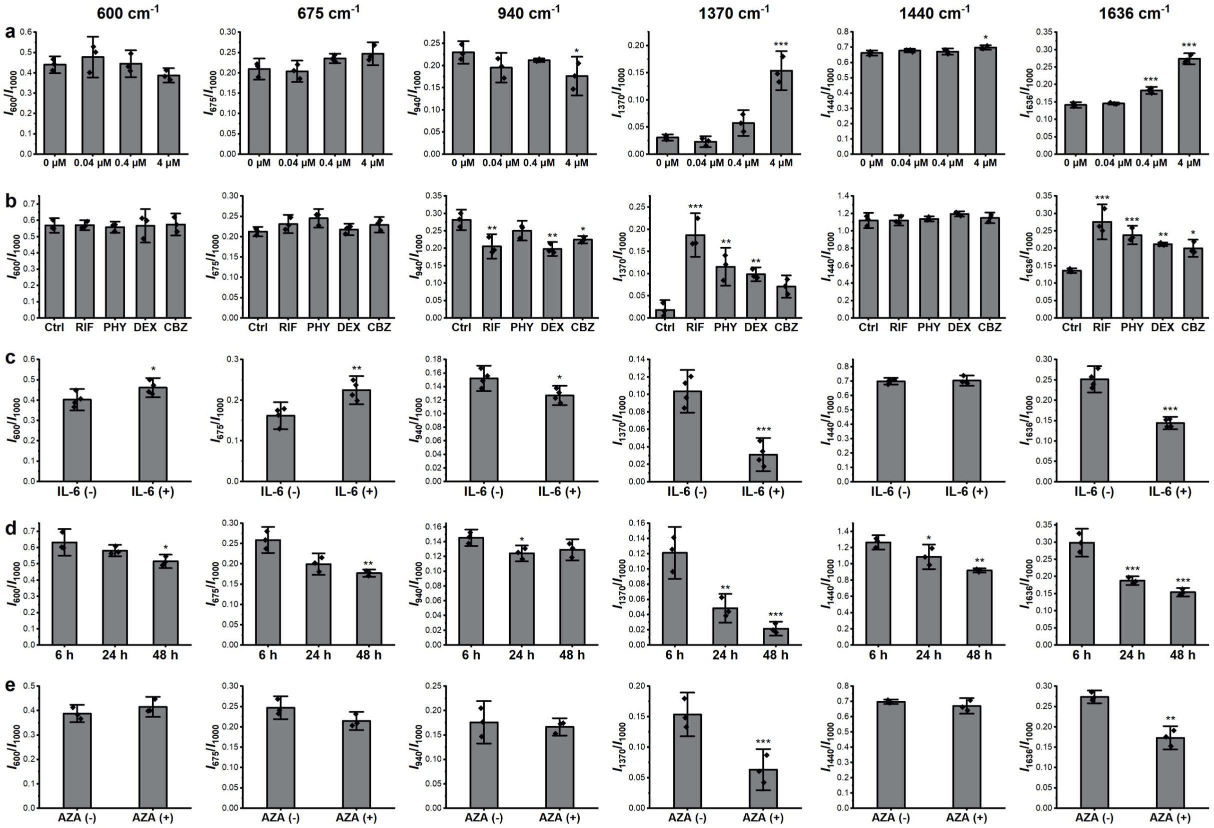

Supplementary Fig. 6 Summary of average Raman intensities at major cellular shifts under various conditions. a Rifampicin gradient. **b** Various inducers. **c** IL-6 treatment. **d** Different culture times. **e** AZA treatment. Under all conditions, 1370 cm^{-1} and 1636 cm^{-1} were more sensitive; the peak at 940 cm^{-1} shows the opposite tendency with respect to inducer treatment (**a** and **b**).

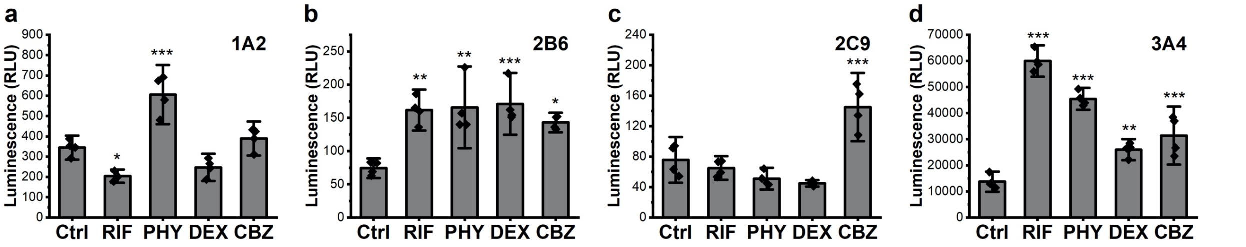

Supplementary Fig. 7 Effects on CYP activity after treatment with various inducers. **a** 1A2 activity. **b** 2B6 activity. **c** 2C9 activity. **d** 3A4 activity.

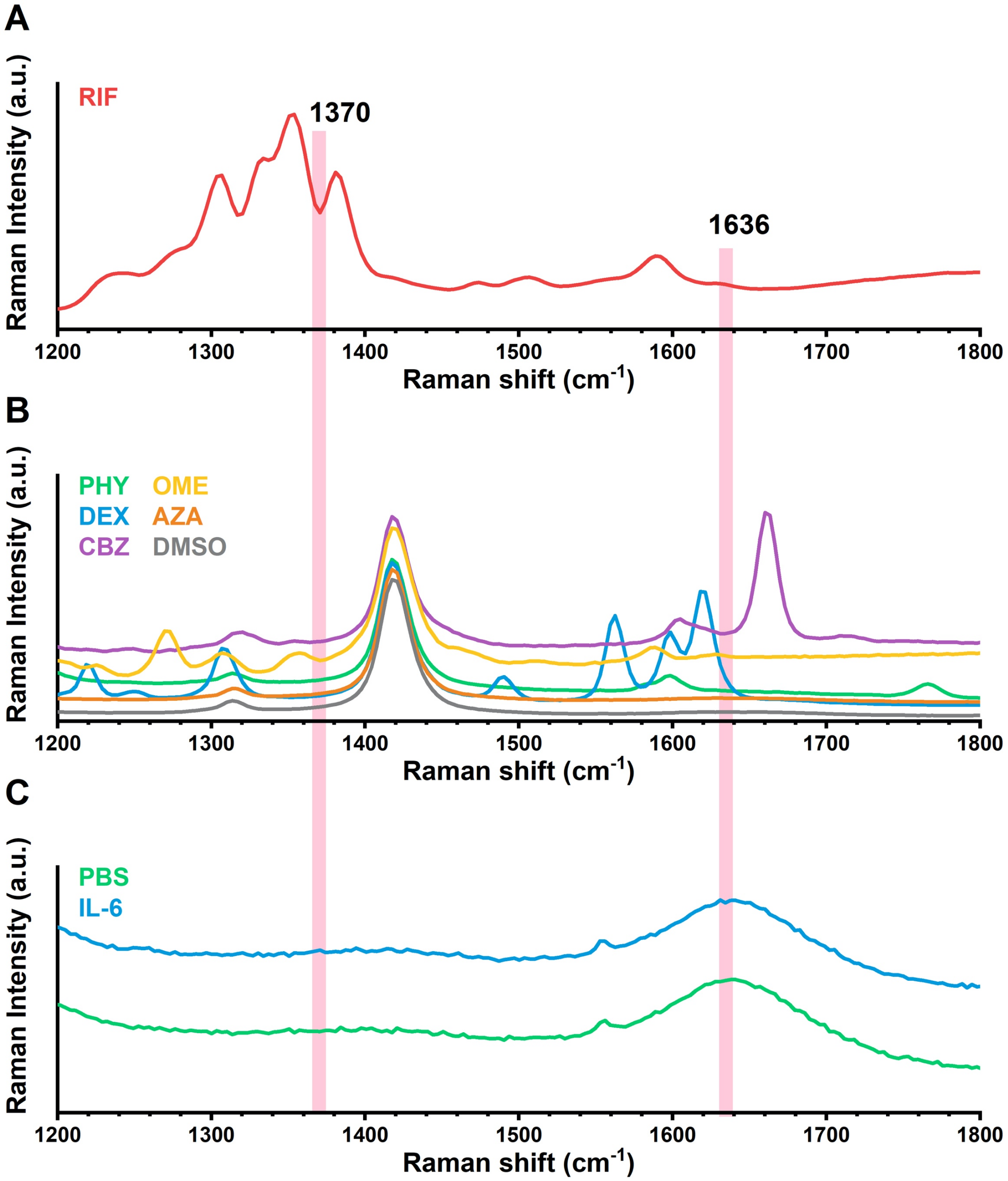

Supplementary Fig. 8 Raman spectra of inducers, down-regulators and inhibitors. a RIF. **b** PHY, DEX, CBZ, OME, AZA, and DMSO (solvent). **c** IL-6 and PBS (solvent). No overlapping Raman peaks at 1370 cm^{-1} or 1636 cm^{-1} were found.

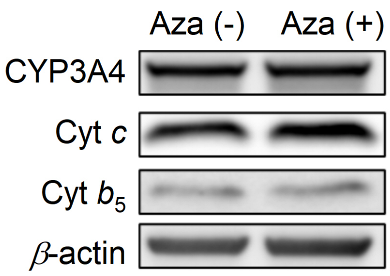

Supplementary Fig. 9 Western blots of hepatic cytochromes with and without AZA treatment. HepaRG cells treated with or without azamulin were collected for western blotting. The amount of CYP3A4 was maintained as well as cyt *c*, cyt b_5 and ß-actin, supporting that the decrease in Raman intensity at 1636 cm^{-1} is due to a change in state, not protein amount.

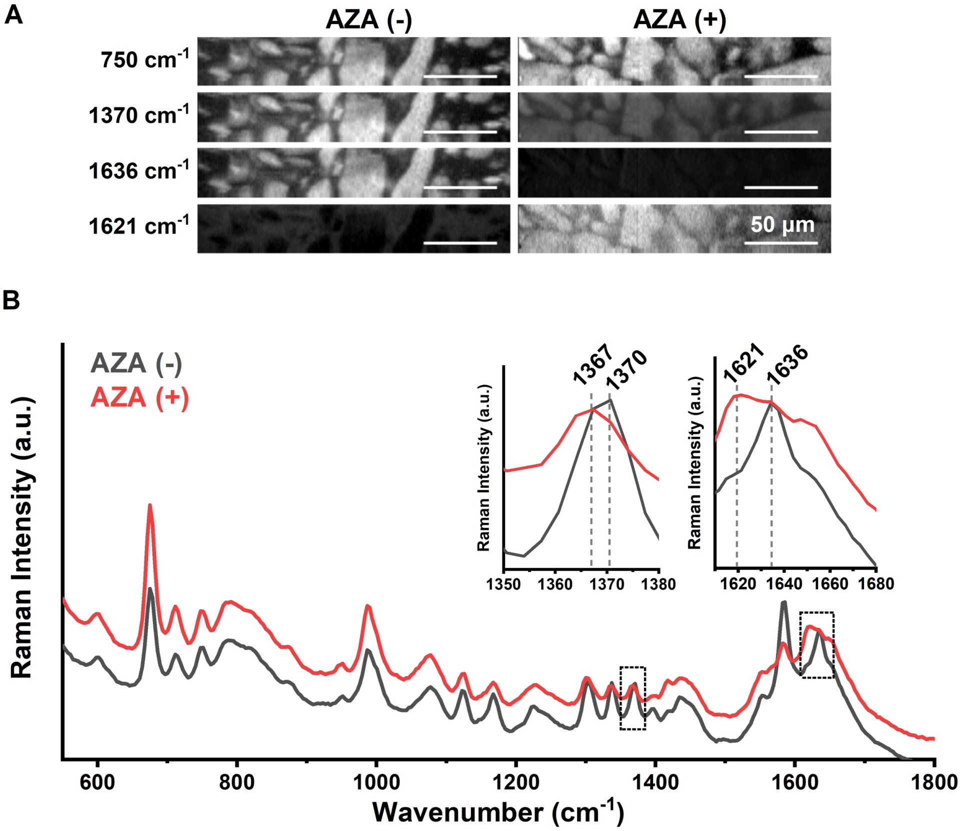

Supplementary Fig. 10 Raman spectra of microsomes and effect of AZA. a Reconstructed Raman images of microsomes at 750 cm^{-1}, 1370 cm^{-1}, 1636 cm^{-1}, and 1621 cm^{-1} with and without AZA treatment. Scale bars, 20 µm. **b** Raman spectra of microsomes treated with and without AZA. The LS marker band at 1636 cm^{-1} decreased after AZA treatment while the HS marker band at 1621 cm^{-1} increased. This indicates that the LS to HS transition occurred due to AZA treatment. On the other hand, the Raman intensity at 1370 cm^{-1} decreased, and a shift to 1367 cm^{-1} was observed; no change in the intensity at 750 cm^{-1} was observed after AZA treatment because 750 cm^{-1} is less sensitive to spin-state transition.

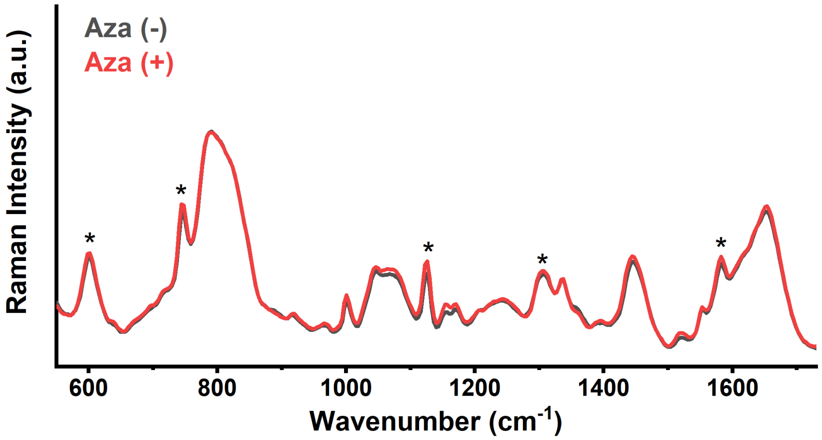

Supplementary Fig. 11 Comparison of HeLa spectra with and without AZA treatment. HeLa cells were treated with 10 μM AZA for 10 min and Raman measurement was performed after treatment. No significant difference was found in the characteristic peaks (asterisks) assigned to reduced cyt *c*.

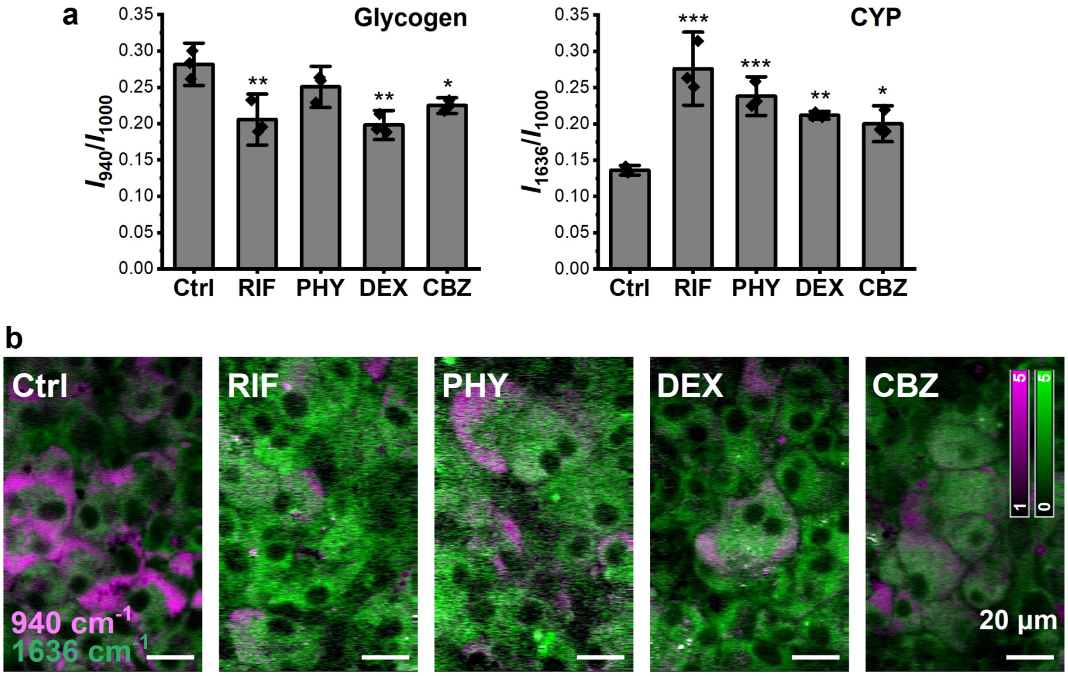

Supplementary Fig. 12 Glycogen and CYP content under treatment with various inducers. Average Raman intensity (**a**) and reconstructed Raman images (**b**) at 940 cm^{-1} (glycogen) and 1636 cm^{-1} (CYP). Inducers promote the expression of CYPs with different levels of glycogen depletion. The simultaneous detection of drug effects on CYP induction and glycogen storage was achieved.

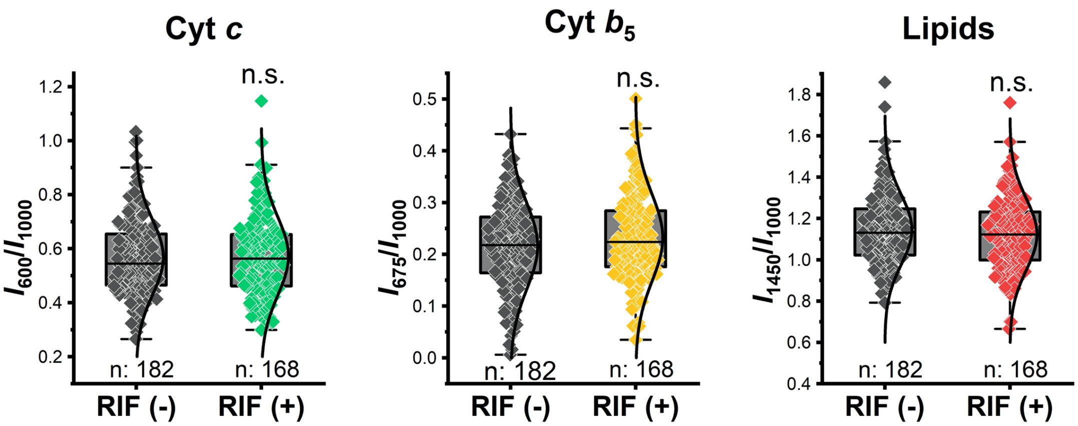

Supplementary Fig. 13 Effect of RIF treatment on Raman intensity of cyt *c*, cyt *b*5, and lipids. Quantitative analysis of Raman intensity at 600 cm^{-1} (cyt *c*), 675 cm^{-1} (cyt *b*5), and 1450 cm^{-1} (lipids) shows no significant difference after RIF treatment. Box plots illustrate the spread within cell populations (center line, median; box, quartiles; whiskers, 1.5× interquartile range). n.s.: $P > 0.05$.

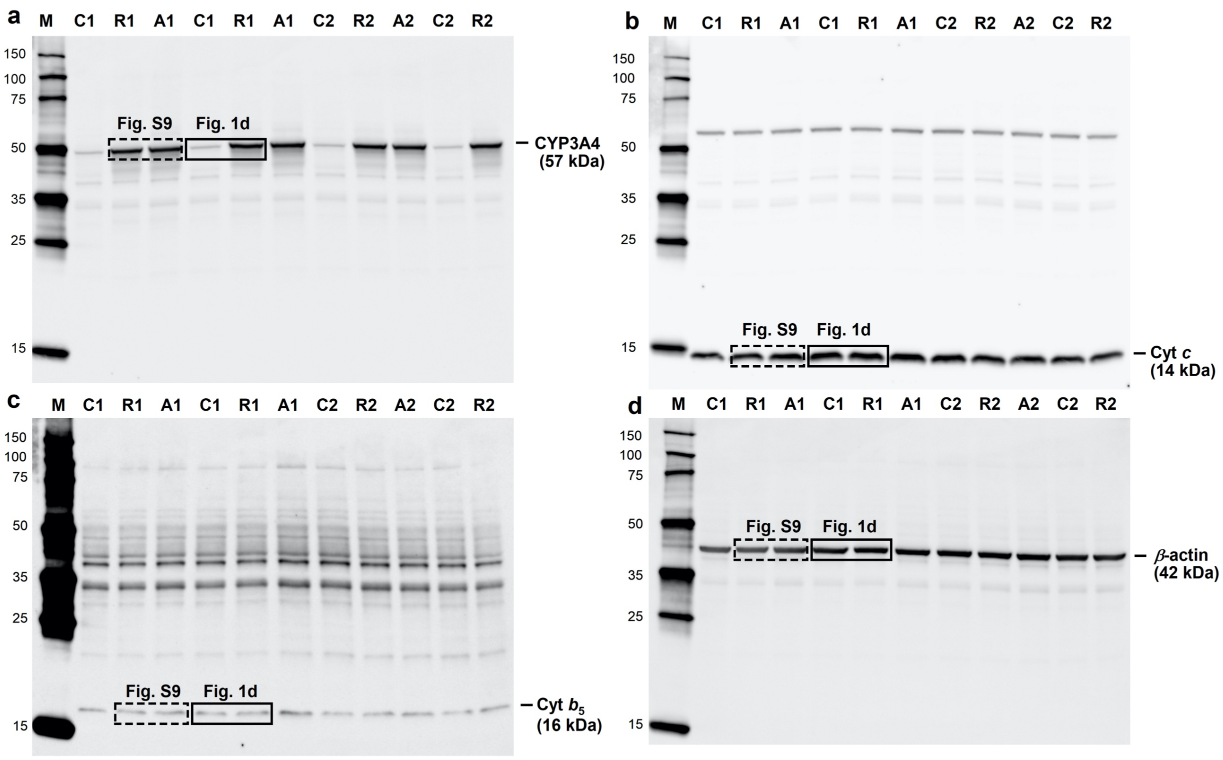

Supplementary Fig. 14 Uncropped western blots used in Fig. 1d and Supplementary Fig. 9. **a** CYP3A4. **b** Cyt *c*. **c** Cyt b_5. **d** ß-actin. C, R, A represent control, RIF treatment, and RIF treatment followed AZA inhibition. Number 1 and 2 represent two independent samples for each condition. Solid frames show the protein bands used in the Fig. 1d. Dashed frames show the protein bands used in the Supplementary Fig. 9.
