# Supplementary material for: Label-free chemical imaging of cytochrome P450 activity by Raman microscopy
Source: Commun Biol. 2022 Aug 22;5:778. doi: 10.1038/s42003-022-03713-1 (PMC9395422; doi:10.1038/s42003-022-03713-1)
Supplement: Supplementary file 3 — Reporting Summary [file 42003_2022_3713_MOESM3_ESM.pdf]

nature portfolio

Corresponding author(s): Katsumasa Fujita

Last updated by author(s): Jun 27, 2022

Reporting Summary

Nature Portfolio wishes to improve the reproducibility of the work that we publish. This form provides structure for consistency and transparency in reporting. For further information on Nature Portfolio policies, see our Editorial Policies and the Editorial Policy Checklist.

Statistics

For all statistical analyses, confirm that the following items are present in the figure legend, table legend, main text, or Methods section.

n/a	Confirmed	
☐	☒	The exact sample size (*n*) for each experimental group/condition, given as a discrete number and unit of measurement
☐	☒	A statement on whether measurements were taken from distinct samples or whether the same sample was measured repeatedly
☐	☒	The statistical test(s) used AND whether they are one- or two-sided<br>*Only common tests should be described solely by name; describe more complex techniques in the Methods section.*
☒	☐	A description of all covariates tested
☐	☒	A description of any assumptions or corrections, such as tests of normality and adjustment for multiple comparisons
☐	☒	A full description of the statistical parameters including central tendency (e.g. means) or other basic estimates (e.g. regression coefficient) AND variation (e.g. standard deviation) or associated estimates of uncertainty (e.g. confidence intervals)
☐	☒	For null hypothesis testing, the test statistic (e.g. *F*, *t*, *r*) with confidence intervals, effect sizes, degrees of freedom and *P* value noted<br>*Give P values as exact values whenever suitable.*
☒	☐	For Bayesian analysis, information on the choice of priors and Markov chain Monte Carlo settings
☒	☐	For hierarchical and complex designs, identification of the appropriate level for tests and full reporting of outcomes
☒	☐	Estimates of effect sizes (e.g. Cohen's *d*, Pearson's *r*), indicating how they were calculated

Our web collection on statistics for biologists contains articles on many of the points above.

Software and code

Policy information about availability of computer code

Data collection	Raman spectra were recorded by WinSpec (Ver. 2.6.24.0). Immunofluorescence images were captured by NIS-Elements (Ver. 3.22.00). PAS staining images were captured by NIS-Elements F (Ver. 5.21.00). Western blots were visualized by Image Lab touch (Ver. 2.4.0.03). Luminescence intensity was detected by Gen5 (Ver. 3.03).
Data analysis	ImageJ 1.53c, OriginPro2022 and MATLAB R2019b were used to analyze the data.

For manuscripts utilizing custom algorithms or software that are central to the research but not yet described in published literature, software must be made available to editors and reviewers. We strongly encourage code deposition in a community repository (e.g. GitHub). See the Nature Portfolio guidelines for submitting code & software for further information.

Data

Policy information about availability of data

All manuscripts must include a data availability statement. This statement should provide the following information, where applicable:

- Accession codes, unique identifiers, or web links for publicly available datasets
- A description of any restrictions on data availability
- For clinical datasets or third party data, please ensure that the statement adheres to our policy

All data necessary to evaluate our conclusions are included in the main manuscript and the supplementary material. All data used in this paper are available from the corresponding author on reasonable request.

Human research participants

Policy information about studies involving human research participants and Sex and Gender in Research.

Reporting on sex and gender	n/a
Population characteristics	n/a
Recruitment	n/a
Ethics oversight	n/a

Note that full information on the approval of the study protocol must also be provided in the manuscript.

Field-specific reporting

Please select the one below that is the best fit for your research. If you are not sure, read the appropriate sections before making your selection.

☒ Life sciences ☐ Behavioural & social sciences ☐ Ecological, evolutionary & environmental sciences

For a reference copy of the document with all sections, see nature.com/documents/nr-reporting-summary-flat.pdf

Life sciences study design

All studies must disclose on these points even when the disclosure is negative.

Sample size	The experiments described in this exploratory study were done for the first time. No pre-specified effect size could be determined a priori. Besides, the hyper-spectral Raman images contain 100800 spectra per image in Fig. 1, 4, 5, and 33600 spectra in Fig. 3. For single-cell based quantitative analysis, around 200 cells per condition were utilized. The spectrum of one individual cell is averaged from several hundreds of spectra. With this much quantity of Raman spectra and cell numbers, we could identify significant difference among conditions.
Data exclusions	Data points that did not provide sufficient signal-to-noise ratio were removed.
Replication	Experiments were replicated at least twice. If not otherwise indicated, all quantitative data describe independent biological triplicates with independent cell seeding and treatments
Randomization	Cells were seeded and cultured at the same condition. Cell dishes were randomly selected for different drug treatment.
Blinding	Investigators were not blinded

Reporting for specific materials, systems and methods

We require information from authors about some types of materials, experimental systems and methods used in many studies. Here, indicate whether each material, system or method listed is relevant to your study. If you are not sure if a list item applies to your research, read the appropriate section before selecting a response.

Materials & experimental systems

n/a	Involved in the study	
☐	☒	Antibodies
☐	☒	Eukaryotic cell lines
☒	☐	Palaeontology and archaeology
☒	☐	Animals and other organisms
☒	☐	Clinical data
☒	☐	Dual use research of concern

Methods

n/a	Involved in the study	
☒	☐	ChIP-seq
☒	☐	Flow cytometry
☒	☐	MRI-based neuroimaging

Antibodies

Antibodies used	Primary antibodies: Mouse anti-CYP3A4 (IF and WB, 1:1000, Sigma-Aldrich, SAB5300118), rabbit anti-human cyt b5 (IF and WB, 1:500, abcam, ab69801), mouse anti-cyt c (IF, 1:200, Santa Cruz, sc-13561), mouse anti-cyt c (WB, 1:200, abcam, ab65311), rabbit anti-ß-actin (1:1000, CST, 4970S)<br>Secondary antibodies: Goat anti-mouse Alexa 488 (IF, 1:200, Invitrogen, A11001), goat anti-rabbit Alexa 594 (IF, 1:200, Invitrogen,

	A11012), HRP-conjugated sheep anti-mouse (WB, 1:10000, Bio-Rad), HRP-conjugated donkey anti-rabbit (WB, 1:10000, Bio-Rad)
Validation	All are validated antibodies by the vendors. Antibody specificity was evaluated using the proper negative and positive controls.

Eukaryotic cell lines

Policy information about cell lines and Sex and Gender in Research

Cell line source(s)	Differentiated HepaRG cells (HPR116) were purchased from Biopredic International.
Authentication	The cell line is not authenticated.
Mycoplasma contamination	HepaRG cells are tested negative for mycoplasma contamintaion by the vendor.
Commonly misidentified lines (See ICLAC register)	No commonly misidentified cell lines were used.
